# Impact evaluation of a school-based oral health program: Kuwait National Program

**DOI:** 10.1186/s12903-019-0895-1

**Published:** 2019-09-02

**Authors:** Aishah Alsumait, Mohamed ElSalhy, Sahar Behzadi, Kim D. Raine, Rebecca Gokiert, Ken Cor, Sabiha Almutawa, Maryam Amin

**Affiliations:** 1 National School Oral Health Program, Ministry of Health, PO Box No 5338, 22064 Salmiya, Kuwait; 2 College of Dental Medicine, University of New England, Portland, ME USA; 3 School of Public Health, University of Alberta, Edmonton, Alberta Canada; 4 Faculty of Extension, University of Alberta, Edmonton, Alberta Canada; 5 Faculty of Pharmacy and Pharmaceutical Sciences, University of Alberta, Edmonton, Alberta Canada; 6 School of Dentistry, Faculty of Medicine and Dentistry, University of Alberta, Edmonton, Alberta Canada

**Keywords:** Children, Caries, Mothers, Knowledge, Oral health quality of life

## Abstract

**Background:**

This study evaluated the relationship between enrolment in a school-based oral health prevention program (SOHP) and: 1) children’s dental health status and oral health-related quality of life (OHRQoL), and 2) mothers’ oral health (OH) knowledge, attitude, practice, and OHRQoL.

**Methods:**

This cross-sectional study, in the Kuwait Capital, included 440 primary school children aged 11 to 12 years and their mothers. Participants were classified into two groups: SOHP and non-SOHP. The SOHP group had been enrolled in the prevention program for at least 3 years: children had twice-a-year applications of fluoride varnish and fissure sealants if needed; mothers had, at least, one oral health education session. The non-SOHP group had negative consents and had not been exposed to the prevention program activities. Dental examinations were performed at schools using portable dental units. Caries experience was determined using the decayed (D/d), missing (M/m), and filled (F/f) teeth (T/t)/surface (S/s) indices. Children’s OHRQoL was assessed using a self-administered validated Child Perceptions Questionnaire 11–14 (CPQ_11–14_). Mothers’ OH knowledge, attitude, practice, and OHRQoL were also assessed. After Bonferroni correction, a *p*-value of less than 0.05 was considered statistically significant for caries experience measures while a p-value of less than 0.013 was considered statistically significant for OHRQoL subscales and mothers’ OH knowledge, attitude, practice, and OHRQoL.

**Results:**

Mean (SD) DT/dt, DMFT/dmft and DMFS/dmfs were 1.41 (1.66), 2.35 (2.33), and 4.41 (5.86) for SOHP children, respectively. For non-SOHP children, the means were 2.61 (2.63), 3.56 (3.05), and 7.24 (7.78), respectively. The difference between the SOHP and non-SOHP was statistically significant (*p* <  0.001). Children enrolled in the program had a higher number of sealed and restored teeth. No significant differences were found in CPQ_11–14_ scores or subscale scores between the two groups. No significant difference in mothers’ OH knowledge, attitude, practices or OHRQoL was found between SOHP and non-SOHP groups (*P* > 0.013).

**Conclusion:**

Enrolment in the SOHP prevention services was associated with a positive impact on children’s caries level with no significant impact on mothers’ knowledge, attitude, practice, or OHRQoL.

## Background

Worldwide, dental professionals struggle to improve public oral health. School-based programs have been established to provide prevention services, including oral health, for school-aged children [1, 2]. These programs can benefit a wide group of children at minimum cost, especially those who are less likely to receive dental care due to economic limitations and limited exposure to fluoride [2]. In fact, using the school as a setting for oral health promotion interventions was recommended in 2002 by the World Health Organization (WHO). Activities in school programs have included oral health education/promotion, supervised tooth brushing, fluoride and fissure sealant application, and/or various treatments. These activities can shape children’s health-related beliefs, attitudes, values and behaviors. In addition, evidence suggests that oral health services in childhood can influence a healthy lifestyle into adulthood [3, 4]. Although school-based oral health education has been found to be effective in promoting oral hygiene and improving oral health knowledge and practices [5, 6], there is a research gap in the literature on the impact of multi-approach preventive programs [1].

In 1983, the Kuwaiti government established the School Oral Health Program (SOHP) in the capital region. The program is a partnership between the Kuwaiti Ministry of Health and the Forsyth Institute, Cambridge, U.S.A., in reaction to the 80% caries rate found in the 1982 national oral health survey [7]. The SOHP was established to provide dental education, prevention, and treatment for children between the ages of 6 and 14 years. These prevention services are offered through mobile clinics at the schools, preventive services, and center-based clinics (See Table 1 for the logic model associated with SOHP). The program provided dental care for approximately 300,000 school children, with 60–70% of these children receiving preventive (general oral health education, tooth-brushing education services, fluoride varnish, fissure sealants) or therapeutic dental care-treatment in 2004 [7].

**Table 1:** School prevention and education logic model

Recourses	Activities	Outputs	Short-term outcomes	Intermediate outcomes	Long-term outcomes
Prevention:					
Prevention team leader Prevention teams: • Dentists • Hygienists • Dental Assistants. Location: schools	Provide fluoride varnish (FL), Fissure sealants (FS)	Children with FL and FS protection	1. Increase proportion of the child population is protected with FL and FS	Lower incidence of decay	Lower incidence of OH related disease Improve OHR-QoL among children
Education:					
Education team leader Education teams: • Dentists • Hygienists Location: Schools	Education sessions at schools, (children, mothers, teachers). Participate in school activities	Knowledgeable teachers, parents and children	1. Increase number of schools in the program 2. Increase number of enrolled children in the program 3. Increased OH knowledge in children, teachers, and parents	Improve OH behavior of children, teachers, and parents	Lower incidence of OH related disease Improved OHR-QoL among mothers’ and children

The Kuwaiti School Oral Health Program has the following broad goals: (1) reduce the prevalence of dental caries among school children by increasing the proportion of caries-free children; (2) reduce the severity of dental caries; and (3) improve the oral health status among this population through oral health education involving parents, teachers and children [7]. Most of the children enrolled in the program receive fluoride varnish applications, and about half of them have at least one sealed tooth. Around 4000 h are spent annually on education, including special sessions conducted for children, parents, and teachers in the schools [7].

Oral health-related quality of life (OHRQoL) reflect subjective indicators of oral health status and its impact on various aspects of life [8]. It is based on information provided by individuals. OHRQoL measures provide essential information when assessing the treatment needs of individuals and populations. They are also important for making clinical decisions and evaluating interventions, services, and public health programs [9, 10]. OHRQoL can be measured using four domains: oral symptoms, functional limitations, social well-being, and emotional well-being. OHRQoL domains are interconnected and affect one another. Dental health status was shown to have a direct impact on children’s overall OHRQoL [11].

Four national oral health surveys were conducted by SOHP in 1982, 1985, 1993, and 2001 to determine the dental health status of children aged 5–15 years. The surveys showed no improvement in dental caries incidence in the population [12, 13]. As these surveys do not differentiate between SOHP enrolled and non-enrolled children, they cannot reflect the effectiveness of the program. The impacts of the SOHP components had not been fully evaluated. Therefore, this study aimed to examine the relationship between enrolment in a school-based oral health program (SOHP) and (1) children’s dental health status and Oral Health-Related Quality of Life, and (2) mothers’ oral health knowledge, attitude, and practice, and OHRQoL.

## Methods

### Study design

This is a cross-sectional study of mother-child dyads from randomly selected public schools at Kuwait Capital School District. The University of Alberta Research Ethics Board (Protocol #0037434), together with the Joint Committee for the Protection of Human Subjects in Research of Kuwait, approved the study protocol. Furthermore, the research was conducted in adherence with the Helsinki Declaration and the STrengthening the Reporting of OBservational studies in Epidemiology (STROBE) guidelines, and the parents/guardians of each participating child granted their written informed consent.

### Participants and setting

The research was conducted for 6 months during the 2013–2014 academic year in the Capital Education/Health Region in Kuwait. Approximately 85% of children residing in Kuwait attend the public schools. Seven schools were randomly selected from a school list provided by the Ministry of Education Research Department. Within these seven schools, children aged 11–12 and their mothers were selected to participate. Participants were classified into two groups; SOHP and non-SOHP. The SOHP participants had been enrolled in the prevention program of the SOHP for at least 3 years. These children received, at least, one oral health education session, two applications of fluoride varnish, and (if eligible) fissure sealants. Non-SOHP participants had negative consents and had not been exposed to the program preventive activities.

A required sample size of 370 participants was calculated based on the number of children in the capital region (16,361 children) using type I error of 0.05, sampling error of 0.05, with a goal to achieve an estimated proportion of children enrolled in the SOHP of 0.5. A 75% positive response rate was expected, so a total of 500 consent forms were distributed.

### Procedure

Trained field assistants gave a 5-min presentation to the children about the study and sent an information letter and consent form home with them for their parents to sign. Once the children’s consent was obtained, field assistants administered structured questionnaires. Clinical examinations were performed at school clinics using fully equipped mobile dental chairs and sterile WHO probes and mirrors.

### Outcome measures

#### Children’s dental examinations

Clinical examinations were conducted at the school clinics to assess the normative needs of the children. The examinations, which were carried out by one calibrated examiner, were carried out in adherence to the oral health criteria recommended by the WHO. The examiner had already used the WHO criteria during the Kuwait National School Oral Health Survey (2013–2014). The examiner was blinded for the SOHP status.

The primary equipment comprised a mobile dental chair, artificial LED light, and a dental unit. High intra-examination and inter-examination consistency (kappa = 0.91 and 0.83, respectively) were shown by the examiner. Evaluations of differences between cavitated and non-cavitated lesions followed criteria outlined in the guidelines of the International Caries Detection and Assessment System (ICDAS) (https://www.icdas.org). Additionally, the Silness-Löe plaque index [14] was used to evaluate the oral hygiene of participating children, while the PUFA index [15] measured the clinical consequences of the participants’ untreated dental caries.

Decayed teeth (DT/dt), missing teeth due to caries (MT/mt), filled/restored teeth (FT/ft), DMF teeth (DMFT/dmft), and DMF surfaces (DMFS/dmfs) indices were used during the examination. Additional indices applied during the examinations included the number of sealed teeth, the number of non-cavitated teeth, the restorative care index (RI) [16] and the plaque index (PI). The PUFA index (for comprehensive oral health examination purposes) was also used.

#### Children’s OHRQoL

To assess each participating child’s oral impacts on function, lifestyle activities, general sense of well-being, and relationship with others [11], the Child Perceptions Questionnaire (CPQ_11–14_), developed in Toronto, Canada by Jokovic et al. [9], was used. The questionnaire measures the following four domains and is reported in subscales: 1) oral symptoms, which are mainly pain-related; 2) functional limitations, such as difficulties encountered while eating and drinking; 3) emotional well-being, such as avoiding smiling or laughing around other people; and 4) social well-being, which involves, for instance, receiving comments from other children about his/her mouth. Overall, the questionnaire has good psychometric properties, including good internal consistency and test-retest reliability. The questionnaire can be self-administered or administered by an interviewer, with only minor differences in results [9]. In the present study, the participating children were given the CPQ_11–14_ self-administered form. The CPQ_11–14_ was translated from the original English version into Arabic, and the translation was validated by Brown and Al-Khayal [17].

In the questionnaire, children were asked to give their oral health a global rating and to describe the impact of their oral health status on their general well-being. The following information was used as a dependent variable in the analysis as a further indicator of OHRQoL. Across the two questions, the response options are: “Excellent” = 0, “Very good” = 1, “Good” = 2, “Acceptable” = 3 and “Poor” = 4 for the global rating; and “Not at all” = 0, “Very little” = 1, “Somewhat” = 2, “A lot” = 3 and “Very much” = 4 for impact on general well-being.

Questions related to perceptions around the impacts of oral health were grouped into the following four domains: oral symptoms (6 questions), functional limitations (9 questions), emotional well-being (9 questions), and social well-being (12 questions). Participants were given five possible response options. The options were Never (0), Once or twice”(1), Sometimes (2), Often (3), and Everyday or Almost Everyday (4) to be applied within a recall period of 3 months. From these responses, domains and overall OHRQoL scores on the questionnaire were calculated by adding up the responses of the items in the domains or the whole questionnaire. Low scores indicated a better OHRQoL.

Prior to administering the questionnaire for research purposes, the Arabic-version questionnaire was given to a small group of participants as a ‘pre-test’. Based on participants’ responses, terms and phrases that were unclear were reworded to make them easier for the children to understand. A total of 118 questionnaires were administered twice (for a grand total of 236), with a 14-day gap between the administration of the initial pre-test and the repeat of the modified pre-test. The kappa scores for the pre-test/pre-retest questionnaires were 0.87–1.0. The reliability results were done with children from two of the selected schools.

For the CPQ_11–14_ questionnaire and each subscale, Cronbach’s alpha was used to quantify internal consistency, while the agreement was measured using the intra-class correlation coefficient of the repeated questionnaires. The item response rate was 100%, with the results suggesting high levels of internal consistency for the questionnaire. As tested by Cronbach’s alpha for the overall CPQ_11–14_ in the sample, reliability was 0.91. Internal consistency coefficients for emotional and social well-being subscales were excellent, measuring 0.83 and 0.81, respectively. However, the alpha coefficient for the functional limitation subscale was acceptable (0.7), but only moderate (0.58) for the oral symptoms subscale. The intra-class correlation coefficient for repeated applications of the measure was excellent at 0.89 (95% CI = 0.76–0.97).

#### Mothers’ knowledge, attitude, practice, and OHRQoL

Mothers were the second target population for the SOHP. According to a previously published report among the Kuwaiti population, mothers were the main caregiver and had a high influence on children’s oral health status [18]. A questionnaire on the mothers’ oral health knowledge, attitude, practice and OHRQoL was used in this study. It was initially prepared in English and translated into Arabic. Then two independent translators, to check for the correctness of the messages conveyed, translated it back into English. Questions included in the questionnaire have been used in previously published studies [19, 20].

In addition to a section on demographics (i.e., age, educational attainment, and number of children), the questionnaire consisted of four sections. The first section included 14 questions that assessed the participants’ oral hygiene knowledge. Questions were about the association between oral bacteria and systemic diseases, transmission of bacteria from parent to child, soft drinks and tooth wear, sugar intake and caries, the frequency of brushing and flossing, type of toothbrush and replacement frequency, dental check-up frequency, and use of fluoridated toothpaste. The second section consisted of six questions about attitudes toward OH including the importance of OH for overall health, the relationship between healthy diet and healthy teeth, the exclusion of unhealthy foods from school premises, the inclusion of oral health topics in the school curriculum, and mothers as oral health educators and role models for their children and students in general. The participants’ oral health-related behaviors (e.g., last dental visit, brushing and flossing frequency, use of fluoridated toothpaste, etc.) were explored in the third section.

The final section asked about the participants’ OHRQoL in three domains: physical, social, and psychological (self-esteem) impairments. The questions asked participants how often oral health problems affected their daily/social activities, and whether the appearance of their teeth caused them to avoid conversation.

### Data analysis

The data were managed and analyzed using SPSS 21.0 software (IBM Corp., Armonk, NY, USA), and data normality were tested using the Kolmogorov-Smirnov test. The children’s dental health measures and CPQ_11–14_ scores, and the mothers’ knowledge, attitude, practice and OHRQoL mean scores were calculated and compared between children grouped by SOHP enrollment. Mean differences according to SOHP enrollment were evaluated by t-test. SOHP and non-SOHP outcomes that were significantly different were further evaluated using Poisson regression analysis that controlled for socioeconomic indicators, such as gender, the number of siblings, mother’s education and mother’s age. The significance level was 0.05. To accommodate for multiple comparisons, the Bonferroni correction was used for every family of variables as follows. A *p*-value of less than 0.05 was considered statistically significant for oral health indicators while p-value of less than 0.013 was considered statistically significant for OHRQoL subscales and Mothers’ knowledge, attitude, practice, and OHRQoL.

## Results

### Characteristics of the studied population

Signed consent forms with respective parent authorizations were returned by 449 participating children, giving a response rate of 88%. Of these 449, nine participants were excluded due to the presence of a systemic disorder which may affect oral health status (as reported by the parents), or due to the child’s uncooperative behavior either during the clinical examination or the administration of the questionnaire.

Four hundred and forty children were included in the final sample, of which 49.3% were male. Approximately half (46.4%) of the children were from families with 2–4 offspring while less than a tenth (7.7%) of the participants had no siblings. Nearly half of the mothers had earned a post-secondary degree and were 39 years of age or younger. Table 2 presents participants’ demographic summary.

**Table 2:** Basic Demographics of participants according to SOHP enrolment

Variable	Number (%)	SOHP *N* = 237	None-SOHP *N* = 203	*P*-value^**^
Children gender				
Male	217 (49.3)	114	103	0.581
Female	223 (50.7)	123	100	
Mother education				
High school or less	94 (21.4)	31	63	< 0.001
More than High school	106 (24.1)	59	57	
College	203 (46.4)	134	69	
Post-College	27 (6.1)	13	14	
Number of children in the family				
Only child	34 (7.7)	20	14	0.01
2–4	204 (46.4)	124	80	
More than 4	202 (45.9)	93	109	
Mother age^a^				
Less than 40	227 (53.0)	122	105	0.92
40 and more	201 (47.0)	109	92	

*SOHP* School Oral Health Program

^**^ Chi-Square test. Significant at the level *p* < 0.05

^a^Total less than 440 due to missing information

### Participants’ OH indicators

Mean (SD) DT/dt was 1.96 (2.24) while the mean DMFT/dmft and DMFS/dmfs were, 2.91(2.75), and 5.71 (6.94), respectively. About 23.9% of the children had a DMFT/dmft of zero. The mean number of non-cavitated carious teeth per child was 2.34 (2.17) while the mean sealed teeth was 1.78 (2.56). The mean restorative index was 0.21 (0.34), the plaque index was 3.59 (1.63), and the PUFA index was 0.31 (0.85). Children’s dental health status according to the demographics was reported in a previous paper [21].

The children enrolled in the SOHP had significantly lower DMFT/dmft (*P* <  0.001), DMFS/dmfs (*P* < 0.001), dt/Dt (*P* < 0.001) scores than non-enrolled children. In addition, they had a higher number of teeth with fissure sealants. The children’s dental health status according to their SOHP enrollment is summarized in Table 3.

**Table 3:** Children’s mean (SD) OH measures and OHRQoL scores according to SOHP enrolment

Variable	Total	SOHP *N* = 237	None-SOHP *N* = 203	*P*-value^*^
Oral Health measures				
dmft/DMFT	2.91 (2.75)	2.35 (2.33)	3.56 (3.05)	< 0.001
dmfs/DMFS	5.71 (6.94)	4.41 (5.86)	7.24 (7.78)	< 0.001
dt/DT	1.96 (2.24)	1.41 (1.66)	2.61 (2.63)	< 0.001
mt/MT	0.28 (0.83)	0.25 (0.80)	0.32 (0.85)	0.391
ft./FT	0.67 (1.19)	0.69 (1.16)	0.64 (1.24)	0.641
Non-cavitated lesions	2.34 (2.17)	2.35 (2.19)	2.32 (2.14)	0.875
Fissure Sealants	1.78 (2.56)	2.36 (2.79)	1.10 (2.05)	< 0.001
Plaque Index	0.89 (0.41)	0.86 (0.40)	0.95 (0.41)	0.019
PUFA	0.31 (0.85)	0.18 (0.51)	0.47 (1.11)	0.01
Restorative Index	0.28 (0.36)	0.33 (0.38)	0.22 (0.34)	0.008
OHRQoL	Total	SOHP *N* = 237	None-SOHP *N* = 203	*P*-value^**^
Total CPQ_11–14_ score	20.72 (16.81)	19.82 (15.46)	21.74 (18.22)	0.253
Oral symptoms	4.26 (3.32)	4.24 (3.03)	4.29 (3.64)	0.862
Functional limitations	5.41 (4.92)	5.23 (4.44)	5.59 (5.43)	0.457
Emotional well-being	5.48 (6.15)	5.15 (5.95)	5.87 (6.37)	0.228
Social well-being	5.33 (6.05)	5.06 (5.97)	5.64 (6.14)	0.321

*SOHP* School Oral Health Program, *D/d* decayed, *F/f* filled, *M/m* missing, *S/s* surface, *T* permanent teeth, *t* primary teeth, *PUFA* Pulp, Ulcer, Fistula, Abscess, *CPQ*_*11–14*_ Child Perception Questionnaire among 11- to 14 year-old children

^*^t-test with Bonferroni correction. Significant at the level *P* < 0.005

^**^t-test with Bonferroni correction. Significant at the level *P* < 0.001

Having adjusted for potential confounding factors (i.e., gender, number of children, mother’s age, and mothers’ OH knowledge) in the Poisson regression analysis, we have found that enrolment in the SOHP was the only predictors for DMFT/dmft, DMFS/dmfs, DT/dt, and number of sealants (Table 4). The prevalence rate for caries/caries experience was about 50% less in children enrolled in the SOHP while the prevalence rate of sealants was 2.8 times those not enrolled in the SOHP.

**Table 4:** Poisson regression analysis final models predicting children OH indicators significantly associated with SOHP enrolments

Variables	PR^a^	*P*-value	95% CI	
			Lower limit	Upper limit
DMFT				
Constant	3.613	< 0.001	2.449	5.330
Enrolment in the SOHP (No/Yes)	0.582	< 0.001	0.510	0.664
DMFS				
Constant	8.120	< 0.001	6.161	10.702
Enrolment in the SOHP (No/Yes)	0.512	< 0.001	0.466	0.561
DT/dt				
Constant	2.728	< 0.001	1.704	4.366
Enrolment in the SOHP (No/Yes)	0.450	< 0.001	0.385	0.526
Number of sealants				
Constant	0.713	0.226	0.412	1.233
Enrolment in the SOHP (No/Yes)	2.825	< 0.001	2.157	3.700

*SOHP* School Oral Health Program, *D/d* decayed, *F/f* filled, *M/m* missing, *S/s* surface, *T* permanent teeth, *t* primary teeth

^a^Controlled for gender, mother’s education, mother’s age and number of siblings

### Children’s oral health-related quality of life

The average (SD) total CPQ_11–14_ score was 20.72 (16.81). The oral symptoms subscale score was 4.26 (3.32); functional limitations score was 5.40 (4.92), emotional well-being score was 5.48 (6.15), and social well-being score was 5.33 (6.05). Nearly 78% of the participating children evaluated their oral health as being excellent or very good, and 5% evaluated it as being fair or poor. The mean overall self-evaluation of the effect of OH on their life was 0.69 (0.95). More than 80% of children reported that their OHRQoL were “not at all or very little” affected, and 5% reported that it affected their life “a lot or very much.” Children’s OHRQoL according to demographics was previously reported [21].

There was no significant difference between SOHP-enrolled and -non-enrolled children in their total CPQ_11–14_ score or subscales (*P* > 0.013) (Table 3).

### Mothers’ knowledge, attitude, practice and OHRQoL

No significant difference in mothers’ OH knowledge, attitude, practices or OHRQoL was found between SOHP and non-SOHP groups (P > 0.013). The mothers’ mean knowledge, attitude, practice, and OHRQoL scores, according to SOHP, are summarized in Table 5.

**Table 5:** Mothers’ mean (SD) OH knowledge, attitude, practices, OHRQoL scores according to SOHP enrolment

Variable	Total	SOHP *N* = 237	None-SOHP *N* = 203	*P*-value^*^
OH Knowledge score	10.04 (1.57)	10.21 (1.46)	9.84 (1.67)	0.019
OH Attitude score	11.63 (0.53)	5.86 (0.39)	5.77 (0.62)	0.077
OH Practices score	13.81 (2.42)	13.97 (2.45)	13.61 (2.37)	0.120
OHRQoL score	0.95 (1.80)	0.78 (1.64)	1.16 (1.96)	0.027

*OH* Oral Health, *OHRQoL* Oral Health Related Quality of Life, *SOHP* School Oral Health Program

^*^t-test with Bonferroni correction. Significant at the level *P* < 0.013

## Discussion

School oral health programs were recommended by the WHO to be a cost-effective approach to reach and promote oral health in school-aged children and to eliminate inequalities in oral health. In the present research, we investigated the effects of oral health education and preventive interventions on the dental health of school-aged children. To assess the effectiveness (impact evaluation), we chose common variables to measure dental caries, oral hygiene, and quality of life. Overall, children enrolled in the program had better dental health (fewer caries lesion) and enrollment in the program was the main predictor of children’s caries level.

The preventive interventions provided by the SOHP were evidenced-based and recommended by WHO [22]. The effectiveness of fluoride has been acknowledged in caries prevention over the past 40 years, and fluoride varnish has been the core of numerous oral health interventions in school children [23, 24]. Fissure sealant has also been recognized as an effective method for prevention of caries on occlusal surfaces in some studies [25]. Our results showed that twice a year fluoride varnish and fissure sealant as part of the school-based prevention activities were effective in reducing dental caries in children.

In Kuwait, all children in the public schools are encouraged to enroll in the SOHP. Our data indicate that enrollment in school-based prevention activities was associated with better dental health. Being enrolled in the SOHP influenced children’s level of dental caries, the severity of the disease, and oral hygiene status. However, the program had no impact on children’s OHRQoL. We previously reported that the level of dental caries had no impact on children’s OHRQoL [21], a result that was inconsistent with Castro Rde et al. [26]. This inconsistency could be explained by participants’ underreporting due to a lack of oral health literacy among Kuwaiti children, an area which needs more research exploration. Further investigation is required regarding the psycho-social impact of dental diseases among the Kuwaiti population.

The second target of the SOHP was the mothers. An appropriate level of oral hygiene practices is often the ultimate outcome of any oral health education/promotion program [27]. Therefore, the intention of Kuwait SOHP for mothers was also improving their oral health practices through enhancing their knowledge and attitude using the Knowledge-Attitude-Practice (KAP) model. Although mothers of children enrolled in the program had better, but not significantly, oral health knowledge, this was not reflected in their practices. SOHP policy-makers may consider adopting different educational and behavior-change models if a change is a target.

The SOHP group had better oral health status than the reported caries prevalence for all schoolchildren in various Kuwaiti governorates [12, 13]. The overall mean of the children’s CPQ_11–14_ was generally better than that in a study undertaken previously in the region [17] and very similar to studies undertaken in other countries [28]. Published reports have shown that the outcome of preventive care provided through different programs varied based on the local context, and more complex interventions tend to have a lower implementation rate [29]. Many countries provide an extensive preventive regimen, including fluoride varnish, fissure sealant, and oral health education [7, 30] that targets only high-risk populations, and thus evaluating a public health program is a context-sensitive process. It is a challenge to capture such a program’s outcomes in a quantitative study [31], because beyond quantitative measure, the context of the program goals, objectives and resources should also be considered. This could be complemented by a process evaluation (i.e., document review) and qualitative data.

The limitations of this study were twofold: the study design and the evaluation variables. Because this was a cross-sectional study, the causal link between intervention activities and dental health indicators cannot be assessed. For instance, we cannot offer conclusive evidence that fissure sealants reduced the incidence of dental caries. Moreover, the SOHP is the main proxy for free-of-charge dental services in Kuwait, and 90% of children receive mainly therapeutic services in the program’s dental center. Due to many factors confounding the relationship between the program activities and the dental health status, this study cannot conclude that the SOHP is an effective program in preventing dental diseases. However, a significant association was detected between enrollment in the school-based prevention intervention and dental health status. Such results could be introduced by selection bias given the assumption that non-SOPH children being essentially non-responders/decliners.

## Conclusions

In conclusion, the Kuwait school oral health program is a comprehensive multicomponent program that had a positive impact on children’s dental health. Children enrolled in the program had lower caries levels. However, no association was detected between the program enrollment and children’s OHRQoL or mothers’ oral health knowledge, attitude, practice, and OHRQoL. Quality assurance and improvement approaches have to be implemented for the program to achieve better outcomes. A mixed-method approach can be used to understand the program’s internal- and external-context and how improvement can be accomplished.

## Data Availability

The patients’ data will not be shared. However, the datasets used and analyzed during the current study are available from the corresponding author on reasonable request.

## Abbreviations

CPQ_11–14_: Child Perception Questionnaire among 11- to 14-year-old children
dmfs/DMFS: Decayed, missing, or filled teeth
dmft/DMFT: Decayed, missing, or filled teeth
dt/DT: Decayed teeth
ft/FT: Filled teeth
mt/MT: Missing teeth
OH: Oral Health
OHRQoL: Oral Health-Related Quality of Life
PI: Plaque index
PUFA: Pulp, Ulcer, Fistula, Abscess index
SOHP: School-based Oral Health Program
STROBE guidelines: STrengthening the Reporting of OBservational studies in Epidemiology
WHO: World Health Organization
